# Abnormal white matter tracts resembling pencil fibers involving prefrontal cortex (Brodmann area 47) in autism: a case report

**DOI:** 10.1186/s13256-016-1020-6

**Published:** 2016-08-26

**Authors:** Ezzat Hashemi, Jeanelle Ariza, Mirna Lechpammer, Stephen C. Noctor, Verónica Martínez-Cerdeño

**Affiliations:** 1 Department of Pathology and Laboratory Medicine, UC Davis, Davis, USA; 2 Institute for Pediatric Regenerative Medicine and Shriners Hospitals for Children Northern California, 2425 Stockton Boulevard, Sacramento, California 95817 USA; 3 Department of Psychiatry and Behavioral Sciences, UC Davis, Davis, USA; 4 MIND Institute, UC Davis School of Medicine, Davis, USA

**Keywords:** Autism, Pathology, Pencil fibers, Cerebral cortex, Prefrontal, Human, Case report

## Abstract

**Background:**

Autism is not correlated with any neuropathological hallmark as the brain of autistic individuals lack defined lesions. However, previous investigations have reported cortical heterotopias and local distortion of the cytoarchitecture of the neocortex in some cases of autism.

**Case presentation:**

Our patient was a 40-year-old white woman diagnosed at an early age with autism and mental retardation. Pencil fibers were present within the prefrontal cortex (Brodmann area 47) and its composition resembled that of the underlying white matter region. Pencil fibers encompassed most of the extent of the cortical grey matter and were populated by oligodendrocytes, astrocytes, and microglial cells, but not by neurons.

**Conclusions:**

Here we report a new cytoarchitectural abnormality that has not been previously described in autism. Future pathological examinations should keep in mind the potential presence of pencil fibers within the prefrontal cortex of cases with autism.

## Background

Autism spectrum disorders (ASD) are defined by a pattern of qualitative abnormalities in reciprocal social interaction, communication, and repetitive interest and behavior. Altered functioning in several areas of the brain underlies the social and cognitive phenotype in autism. Regions of the brain in which alterations have been identified include the prefrontal cerebral cortex. Autism is accompanied by altered patterns of connectivity in the adult brain and impairments in brain development including cell generation and migration during prenatal cortical development [1–3]. However, the classical symptoms of autism are not correlated with characteristic neuropathological hallmarks as the brain of autistic individuals lack defined lesions. Previous investigations have reported subcortical, periventricular, hippocampal, and cerebellar heterotopias detected in the brains of 30 % of autistic individuals [4]. Multifocal cerebral dysplasia resulted in local distortion of the cytoarchitecture of the neocortex in four brains (31 % of the examined brains), the entorhinal cortex in two brains (15 %), the hippocampus in four brains (31 %), and the dentate gyrus in two brains (15 %) [4]. These alterations included focal patches of abnormal laminar cytoarchitecture and disorganization of cortical neurons in prefrontal and temporal cortical tissue in children with autism [5]. Here, we show for the first time to the best of our knowledge, the presence of a white matter abnormality resembling pencil fibers in Brodmann area (BA) 47 in a case of autism.

## Case presentation

We report the case of a 40-year-old white woman diagnosed with autism whose clinical features included qualitative abnormalities in reciprocal social interaction and communication, and restricted repetitive and stereotyped patterns of behavior together with mental retardation, as is common in autism [6]. Her clinical history (AN07770) was collected by the Autism Tissue Program (ATP) through a parental interview using the Autism Diagnostic Interview - Revised (ADI-R). Later the ATP collected her brain. The ATP has transitioned to a new autism brain network called Autism BrainNet. Summarizing, our patient was a full term baby who weighed 2.95 kg (6 lb 8 oz). Her physical development is described as being normal. She was diagnosed at an early age with mental retardation. Her language was significantly delayed. No signs of convulsions, vision or hearing problems, or neurological abnormalities were diagnosed. She demonstrated apparent adherence to non-functional routines and several sensory aversions to loud noises. She was medicated with Vistaril (hydroxyzine), Decadron (dexamethasone), and Demulen (ethinyl estradiol). Her maternal family history is notable in that her grandmother had emphysema. Her paternal family history is significant in that her father died at age 53 due to atherosclerosis after undergoing two bypass surgeries. Her magnetic resonance imaging (MRI) demonstrated moderate cerebral and cerebellar atrophic changes, and extensive low intensity in her substantia nigra and basal ganglia bilaterally, probably secondary to iron deposition. At age 38 she experienced mood swings, was leaning to the left and walking on her right toe with her right arm curled in. By the end of that year she was unable to walk and was confined to a wheelchair. At age 39 years she was diagnosed with pantothenate kinase-associated neurodegeneration (PKAN), because of the spasticity in her limbs and feet. She experienced rapid and severe regression of her motor skills and ability to speak and died at age 40 due to respiratory arrest. No pathology report was obtained. Her brain was very small (890 grams).

We analyzed prefrontal tissue from areas BA45, 46, 47, and 9. No obvious modification in cell size or density was observed. Of these areas, only BA47 presented with abnormal white matter islands extending into the cortical grey matter. The rest of the brain tissue from this patient was distributed to other research groups. We are not aware of any additional report of pathology in this case. Publications reporting on this case included that by McKavanagh *et al*. that reported on the temporal cortex (BA40, 41, and planum temporale) and the orbitofrontal cortex (BA11) [7].

**Fig. 1:**
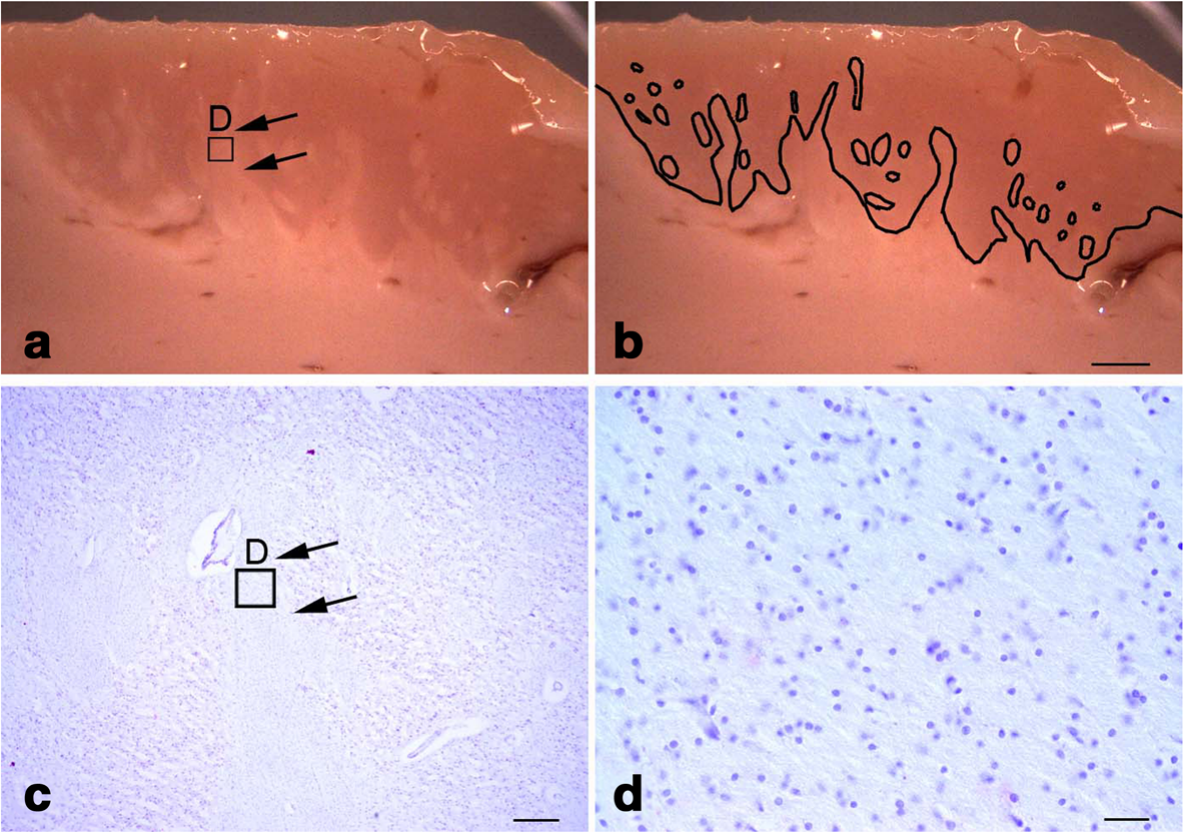
**a** A block of cerebral cortex tissue isolated from Brodmann area 47. The arrows point to one of the pencil fibers that prominently extend into the cortical grey matter. The *inset* labeled ‘D’ is shown at higher power in panel (**d**). **b** Same image shown in panel (**a**) in which the outline of the pencil fibers is delineated with a black line. **c** A section of tissue obtained from the block stained for Nissl substance. Arrows in panel **c** point to the location of the pencil fiber that is also indicated with arrows in panel **a**. The *inset* labeled ‘D’ is shown at higher power in panel (**d**). **d** Higher power magnification of the inset shown in panels (**a**) and (**c**). This image shows the histology within the pencil fibers. Glial nuclei are observable in the Nissl, but not neurons. Scale bars: **a**–**c** 600 μm; **d** 150 μm

**Fig. 2:**
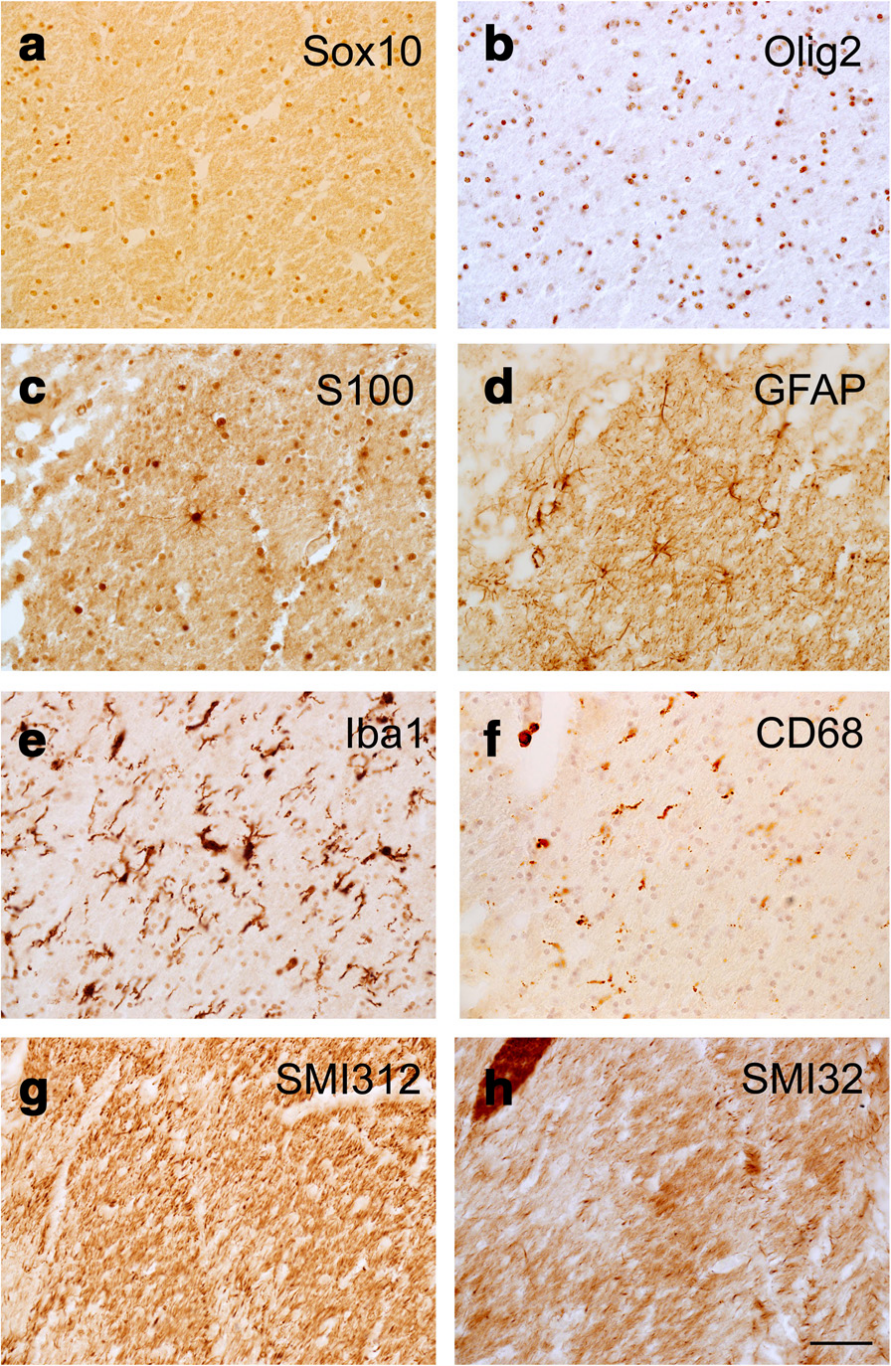
Immunostaining with cell-specific markers in tissue sections that were adjacent to the section shown in Panel 1c. The images were taken within the pencil fibers indicated by the insets in Panels 1a and 1c, and shown in Nissl staining at higher power in Panel 1d. **a**–**f** Markers for glial cells show the presence of glial cells within the cortical pencil fibers: Sox 10 and Olig2 for oligodendrocytes, S100 and GFAP for astrocytes, Iba 1 for microglial cells and CD68 for activated microglia. **g**, **h** Axonal neurofilament 312 and neurofilament H non-phosphorylated 132 demonstrate the presence of axonal fibers within the pencil fibers. Scale bar: 150 μm

Our patient presented with abnormal white matter extensions and islands into the cortical grey matter in BA47. These white matter regions resembled the pencil fibers of the striatum [8] and therefore we referred to them as “pencil fibers.” Cortical pencil fibers have never been described in autism. Pencil fibers encompassed most of the extent of the cortical grey matter, some including all layers from VI to II, but did not extend into layer I (Fig. 1). We performed 14 μm coronal sections of BA47 area and immunostained it with specific cell type markers. We found that oligodendrocytes (SRY (sex determining region Y)-box 10 and oligodendrocyte transcription factor 2; Sox10+ and Olig2+), astrocytes (S100+ and glial fibrillary acidic protein; GFAP+), and microglial cells (ionized calcium-binding adapter molecule 1; Iba1+) were present within the pencil fibers. Some of the microglia were immunopositive for CD68, indicating that they were activated cells (Fig. 2). However, neurons were not present. Pencil fibers  were rich in axonal neurofilament 312 (SMI312+) and neurofilament H non-phosphorylated 132 (SMI32+) fibers. Overall, the cellular composition within the cortical pencil fibers resembled that of the underlying white matter region. We found b-amyloid deposits and tau+ neurofilaments, which are typical of neurodegenerative diseases including PKAN, within the cortical grey matter but not within the white matter pencil fibers. Iron deposits were not detected.

## Discussion

The formation of cortical pencil fibers in this patient most likely arose from abnormal cortical development rather than from a neurodegenerative process. We did not detect any atypical microscopic feature common to neurodegenerative disorders within the pencil fibers such as neuritic plaques and neurofibrillary tangles, dysplastic neurons and/or balloon cells. Therefore, we conclude that pencil fibers in this patient were most likely the product of altered prenatal cortical development linked to autism, rather than the later manifested pathological consequence of PKAN.

The presence of pencil fibers within the grey matter of the cerebral cortex disrupts cortical cytoarchitecture, occupying a position in the cortex that would otherwise be populated by excitatory and inhibitory neurons and glial cells. The abnormal laminar cytoarchitecture and cortical pencil fibers could represent a form of cortical dysplasia resulting from defects in cellular migration during prenatal cortical development. Defects in the radial migration of excitatory cortical neurons can produce defects as observed here. In addition, defects in the tangential migration of inhibitory cortical interneurons could also impair the regional position of these cells and disrupt proper formation of the cortex. Dysregulated patterns of progenitor cell division (radial glial cells and/or intermediate progenitor cells) could also modify the final laminar destination of neurons [9–11]. Alternatively, it is possible that severely impaired axonal guidance disrupted white matter development in this cortical area. Accordingly, 88 % of high-risk genes for autism have been found to influence neural induction and early maturation of newly born cells [2, 12].

## Conclusions

Overall, we show a new cytoarchitectural abnormality, cortical pencil fibers, not previously described in the cerebral cortex of an autistic individual. Future pathological examinations should keep in mind the potential presence of pencil fibers within the prefrontal cortex of cases with autism.

## Abbreviations

ADI-R, Autism Diagnostic Interview - Revised; ASD, autism spectrum disorders; ATP, Autism Tissue Program; BA, Brodmann area; GFAP, glial fibrillary acidic protein; Iba1, ionized calcium-binding adapter molecule 1; MRI, magnetic resonance imaging; Olig2, oligodendrocyte transcription factor 2; PKAN, pantothenate kinase-associated neurodegeneration; S100, protein of low molecular weight characterized by two calcium-binding sites that have helix-loop-helix (“EF-hand type”) conformation; SMI312, axonal neurofilament 312; SMI32, neurofilament H non-phosphorylated 132; Sox10, SRY (sex determining region Y)-box 10
